# Understanding social work’s role in abortion care: A comprehensive scoping review

**DOI:** 10.1371/journal.pone.0320260

**Published:** 2025-04-24

**Authors:** Preetika Sharma, Julie L. Halverson, Yoonhee Lee, Sarmitha Sivakumaran, Cam Bautista, Hajar Seiyad, Ayla Arhinson, Temulun Bagen, Gajathree Ananthathurai, Stephanie Begun

**Affiliations:** 1 Factor Inwentash Faculty of Social Work, University of Toronto, Toronto, Ontario, Canada; 2 University of Toronto Libraries, University of Toronto, Toronto, Ontario, Canada; Delta State University, NIGERIA

## Abstract

**Background:**

Social workers have played a crucial role in abortion care for decades, addressing clients’ diverse abortion care needs. Social workers have ethical duties to provide unimpeded access to information and supports to clients, in pursuit of social justice. This scoping review comprehensively synthesizes published literature on social work’s role in abortion care.

**Methods:**

The review adhered to Arksey and O’Malley’s five-stage framework. The following databases were searched for literature published between 1973–2023: PsycINFO, MEDLINE, CINAHL, Social Work Abstracts, Social Services Abstracts, and Scopus. Search results were uploaded to Covidence for de-duplication and screening. Out of 2,980 articles screened in title and abstract review, followed by full-text review, 78 articles were included.

**Results:**

A majority the 78 studies (*n* = 67) were set in the United States. The remainder were from the United Kingdom, Australia, Canada, New Zealand, and two studies involved multiple countries. Six themes emerged to summarize the literature base: (1) Social workers’ attitudes regarding abortion and abortion-seekers; (2) Abortion stigma and barriers in social work; (3) Social work and reproductive justice; (4) Social work and ethical considerations regarding abortion; (5) Social work and abortion policy and advocacy; and (6) Social work and family planning: roles, approaches, and practice frameworks.

**Discussion:**

Social workers have long played intrinsic roles in abortion care, including counseling, providing access to concrete supports and mitigating barriers to abortion care, and abortion rights advocacy. Social justice and professional ethical commitments underpin social work’s role in abortion care. However, existing gaps in abortion education, training, and practice frameworks are impeded by the dynamic legal landscape and stigma attached to people seeking abortion care. Reproductive justice offers a framework, that overlaps with the field’s social justice tenets, to raise consciousness among social work students, professionals, and researchers, and to address gaps in abortion education, training, and scholarship.

## Introduction

Globally, around 121 million unintended pregnancies occur annually, out of which 61% result in abortion [1]. These numbers emphasize the importance of providing appropriate and accurate health information and counseling to abortion seekers, when needed, to ensure high-quality abortion care [2]. Involving a wide range of healthcare workers in abortion care is one of the many ways recommended by the World Health Organization (WHO) to address this issue [2]. The feasibility of expanding the abortion care workforce has been made more possible with the increased availability of mifepristone and misoprostol which enable the provision of safe and non-invasive medical abortion. In some settings, such abortions can now be provided not only by physicians, but also by professionals such as nurse practitioners and midwives. This expands options for abortion beyond traditional surgical methods and mitigates the specialized training required to administer surgical abortions; in doing so, this also reduces challenges associated with the shortage of skilled healthcare professionals while facilitating greater affordability of abortion care [2].

In addition to providing abortion care, supporting clients through abortion counseling and referrals requires professional training [3]. Research notes that patients’ information-seeking encounters with professionals either enable or impair the autonomy of pregnant persons’ decision-making, in particular, who are seeking abortion information and resources [4]. A core principle of social work practice is to actively support clients as they seek information, unimpeded access to resources and services, and to facilitate client self-determination in all decision-making. Social workers are positioned to play important roles in providing abortion information and referrals. Though social workers are present in countless community-based and institutional settings, they may be somewhat overlooked as a central part of the abortion care workforce, as they traditionally may not be referred to as medical healthcare providers (compared to nurses, midwives, and doctors) but are nonetheless core members of interdisciplinary allied health teams. This scoping review thus explores and synthesizes the literature base regarding the diverse and integral roles that social workers ultimately play in abortion care, also examining intersections of how abortion is discussed in social work practice, research, advocacy and policy, and education.

Social workers are potentially competent in serving clients looking for medically accurate abortion options information, and ultimately, access to abortion [5]. Social workers are commonly part of interdisciplinary healthcare teams, and their extensive training in clients’ holistic social, psychological, and biological needs enables them to make essential contributions to such settings [6]. Deep commitments to and experience working with equity-deserving groups make social workers key members of healthcare teams, as they enhance coordination, accessibility, and safety for service-seekers [7]. Social workers also work in many settings outside of the healthcare system, including schools, children’s aid societies and child welfare, gender-based and family violence shelters, homeless shelters and housing supports, harm-reduction sites, non-profit and nongovernmental organizations (NGOs), the justice system, and community-based organizations; moreover, their work is guided by a well-defined Code of Ethics with imperatives to assist marginalized groups without judgment or stigma [8]. Social workers routinely address individuals’ emotional needs by providing psychoeducation, connecting to unmet non-medical services based on their knowledge of systems navigation, and addressing instrumental and financial needs by making clients aware of available supports [9]. Social workers strive toward empowerment, self-determination, and ultimately, work in pursuit of social justice, by, with, and for clients and client systems [10].

Published research includes numerous relevant articles articulating social work’s connections to abortion care. Social work scholars have emphasized use of the framework of reproductive justice to advocate for abortion rights in social work practice [11–14]. Researchers have further underlined that the very essence of social work’s Code of Ethics is intrinsic to the core principles of reproductive justice [15]. Further, there have been formal statements issued that specify social work’s professional stance in support of unimpeded access to abortion [16,17]. However, despite social work’s professional commitment to abortion rights and self-determined decision-making, coupled with social work’s omnipresence in the abortion care workforce, prior research has noted that abortion has received inadequate attention in social work education [18–21]. Suggestions have been made that social work curricula should further emphasize broader reproductive rights and relevant policies, resource referrals, and advocacy skills to more adequately train social work students who are seeking to most optimally advance human rights and client self-determination through their social work practice [13,19,21,22].

A few studies have assessed the attitudes and beliefs of social workers and social work students toward abortion [20,23–25]. Research has shown that social work students rarely have discussions on the topic of abortion in their classroom settings, with many receiving no training in abortion care; in addition, most do not have knowledge of medical abortion [23]. Research also notes that social workers’ political, personal, and religious beliefs regarding abortion may serve to influence their approaches to service provision, which in some cases, may be at odds with their ethical responsibilities to carrying out duties per the social work Code of Ethics [20,23]. International bodies of social work similarly ascertain the right to abortion care and define the ethical obligation of social workers to respect abortion-seekers’ autonomy while providing abortion-related information and referrals [26], but social work curricula do not overtly emphasize training students in this area [24]. Research also indicates that social workers are under-trained in issues and policies related to reproductive and sexual health, more broadly, and lack fundamental knowledge of available resources across dimensions such as contraception, sexually transmitted infections, pregnancy, parenting supports, and more [20,22,23].

### Scoping review objectives

Accordingly, we found it important to conduct a scoping review to understand and synthesize the literature base to explore the following research question: what is social work’s role in abortion care? No prior scoping review has been conducted, to our knowledge, on this specific topic. This scoping review will aid in identifying key gaps and emerging priorities for training and continuing professional development that could be developed and offered to social work students as well as social workers engaged in the field, also indicating opportunities and implications for further social work research, practice, policy, and advocacy efforts.

## Methods

### Eligibility criteria

Relevant articles were assessed against the following inclusion criteria: (1) the words social work and abortion (inclusive of search terms, respectively) are used in the title or abstract; (2) any dimension of social work (e.g., practice, research, education, policy and advocacy) are discussed in some relationship to abortion, or related topics (e.g., family planning, reproductive health and rights, choice) under which abortion is most often grouped (please refer to S1 Appendix for more detail regarding such search terms); (3) Study conducted in or study context includes the United States, Canada, United Kingdom, Australia, or New Zealand, as the social work profession, though nuanced, is overall congruent across these geographies; (4) Studies conducted from 1973 to present were eligible, marking the timeline from which Roe v. Wade was decided in the United States, a landmark and globally-visible case resulting in abortion becoming constitutionally legal in the U.S.; (5) Studies completed in English and French language were included, to respond to the official languages of the countries covered in the review; and (6) in addition to peer-reviewed literature, grey literature was also included. If the above-mentioned criteria were met, studies were deemed eligible for the review.

### Information sources and search strategy

To identify published and unpublished literature, the following electronic databases were searched: PsycINFO (Ovid), MEDLINE (Ovid), CINAHL (EBSCO), Social Work Abstracts (Ovid), Social Services Abstracts (Proquest), and Scopus. The search was limited to literature published after 1973. No other limits were applied. To supplement the database searches, we also manually searched key journals and the reference lists of relevant articles, in addition to browsing targeted social work websites.

A pilot search strategy was developed by a social work librarian (YL), in collaboration with the project team, in PsycINFO (Ovid) using both textwords and American Psychological Association (APA) thesaurus terms, related to the concepts of social work and abortion. The search strategy was peer-reviewed by another librarian using the Peer Review of Electronic Search Strategies (PRESS) guidelines. The finalized search strategy (S1 Appendix) was then translated into other databases, using subject headings where applicable.

The methods for this scoping review followed the five-stage framework established by Arksey and O’Malley [27] and further updated by Levac et al. [28]. These five stages include: (1) identifying the research question; (2) identifying relevant studies; (3) study selection; (4) charting the data; and (5) data summary and synthesis of results. This review adhered to PRISMA-ScR reporting guidelines [29]. Though scoping reviews are not eligible for PROSPERO registration often used for systematic reviews, for transparency and rigour, we registered our study protocol on Open Science Framework on July 20, 2023 [18].

### Study records and data collection process

**Fig 1:**
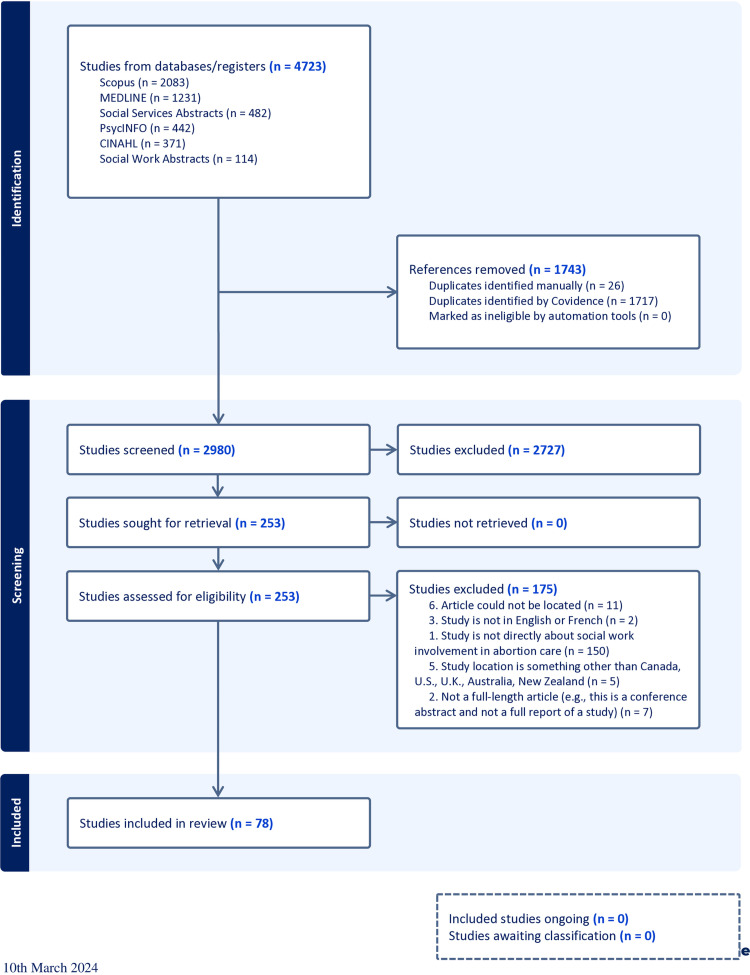
PRISMA Flow Diagram. This PRISMA Flow Diagram shows the systematic process we followed to include papers captured by our search.

Search results were uploaded to the software Covidence for de-duplication and screening. The search and study selection process included two levels of screening and commenced on August 16, 2023: (1) a title and abstract review; and (2) a full-text review. For the first level of screening, seven research team members, including one post-doctoral Research Associate (PS) and six student Research Assistants (GA, AA, TB, CB, HS, SS), each of whom is working under the supervision of the Principal Investigator (“PI” SB), independently reviewed abstracts. Each abstract was reviewed by at least two team members, with votes provided for whether to include or exclude from the study based on the contents of the article’s abstract and title. In cases in which there were conflicts, the PI (SB) reviewed these abstracts after engaging in team discussion regarding differences in rationale provided for inclusion or exclusion. The PI (SB) then provided a final inclusion/exclusion vote. In the second, full-text review stage, the broader inclusion criteria (above) then guided whether to include or exclude studies that preliminarily advanced through the abstract review process, ensuring that the articles chosen in fact did adequately and accurately focus on social work and abortion. This process was again adjudicated by two research team members who provided independent votes regarding inclusion or exclusion, with conflicts again being decided on by the PI (SB) in consultation with the team regarding their review rationale. The results of the search and study inclusion process are presented in a Preferred Reporting Items for Systematic Reviews and Meta-analyses extension for scoping review (PRISMA-ScR) flow diagram [29] (see Fig 1).

### Data analysis and synthesis

Included studies then underwent data extraction. All articles included in the scoping review were organized in a Microsoft Excel spreadsheet by postdoctoral researcher and lead author of this review (PS). Identified themes were charted to present the articles’ content as they relate to the scoping review research questions. As can be seen on S1 Table, charting categories included: authors, publication title and year, study aims and objectives, study design and methods, study location and context, social work area (e.g., practice, education, policy and advocacy, research), relevant findings, and study’s usefulness to review. Following Levac et al. [30], the team reviewed the spreadsheet and identified all emerging themes and connections between social work and its role in abortion care. Data utilized in this study were publicly available as this is a scoping review using content that is considered open to public domain.

We provide our synthesis of the results, as follows; this scoping review reports on our charted results and thematic implications across social work practice, education, policy and advocacy, and research. We will also share the findings from this study through peer-reviewed conference presentations and other knowledge translation activities in venues that are also likely to reach social workers and allied professionals engaging in abortion care.

### Dissemination and ethics

This scoping review will help guide social work students and educators, practicing social workers, healthcare and community-based organizations, policy-makers, advocates, and researchers. This study is part of a larger project that aims to curate resources to improve healthcare and other frontline service providers’ expertise in providing de-stigmatized and contextually appropriate abortion care for populations facing intersecting systemic barriers, and similarly, to reduce gaps in information and resources available for equity-deserving populations. All project outcomes, including this scoping review, aim to support a diverse array of healthcare and frontline service providers by improving their skill-sets to assist those seeking abortion care, while reducing barriers that make abortion services less accessible and appropriate. Research ethics approval for this review is not necessary, as we used publicly available sources to collect data, and no data were collected from human subjects.

## Results

### Study characteristics

The 78 included studies were published between 1973 to 2023. The majority of the studies, 67 in total, were set in the United States. Four studies were based in the United Kingdom, two in Australia, two in Canada, one in New Zealand, and two studies involved multiple countries—one focused on the U.S., U.K., Ireland, and South Africa, and the other included Australia, the Republic of Ireland, and New Zealand. For these, we opted to include the two multi-national studies into the search, but then only extracted pertinent findings and insights that reflected countries that were specifically part of our search and inclusion parameters. We opted to do this so that we would not miss important nuances within the countries of focus in our review, while further diversifying the resulting sample of articles included. The articles were dispersed across topical areas of education (7), policy (18), practice (27), and research (26). Our results included 28 conceptual/thought pieces, 24 quantitative studies, 12 qualitative studies, 3 program or practice development articles, 2 mixed methods studies, 3 clinical chart reviews or case examples, 1 literature review, 1 curriculum review, 1 editorial,1 policy analysis, 1 commentary, and 1 typology development.

### Emerging themes

Six primary themes emerged from the scoping review, including: (1) Social workers’ attitudes regarding abortion and abortion-seekers; (2) Abortion stigma and barriers faced by social workers; (3) Social work and reproductive justice; (4) Social work and ethical considerations regarding abortion; (5) Social work and abortion policy and advocacy; and (6) Social work and family planning roles, approaches, and practice frameworks.

#### Attitudes regarding abortion and abortion-seekers.

Social workers’ attitudes toward abortion were discussed in 15 studies [5,20,23–26,30–40], encompassing assessments of such attitudes held by social work students [5,20,23,25,33,34,36], faculty [34], and social workers practicing in the field [24,26,30–32,35,37]. Five of the studies further integrated or compared social workers’ abortion attitudes to those of other health professionals such as nurses [30,31,34,35,37]. All of these studies were set in the United States and are further illustrated as follows.

Studies examining attitudes most frequently highlighted the abortion attitudes and opinions of social work students. In a nationwide survey of 504 social work students in the U.S., the majority (63.1%) strongly endorsed abortion rights, but a notable percentage (22.8%) opposed abortion legality and (20.6%) specifically opposed abortion after 20 weeks of gestation. The findings also revealed infrequent classroom discussions on abortion, with 70.6% expressing insufficient knowledge of abortion policies [23,25]. A web-based survey of 116 students conducted by Ely found that 49% of participants would not recommend someone for an abortion, 41% were unsure about abortion legality in their State, and higher religiosity showed significant associations with reduced acceptance of abortion, as well as lower likelihoods of making abortion referrals. The study highlighted the potential negative impact of a social worker’s religious beliefs on the profession’s fundamental principle to support client self-determination [26]. Witt found in a survey of 626 students, 68.1% supported that abortion should be legal and 16.3% believed that abortion should be illegal. Only 7.2% of participants in the sample indicated regular discussions of abortion in social work classrooms, and a mere 2.7% received training on the topic during field placements [36]. Winter et al. reported on survey data collected from 443 MSW students and found that students’ anti-abortion attitudes are significantly associated with indicating reluctance to assist clients in reproductive health decisions with which they disagree [24].

Examining and comparing attitudes among social workers and nurses in their 1970s study of 419 social workers and 158 nurses, Hendershot et al. discovered a notable contrast. While social workers exhibited a higher favourability towards abortion services for the economically disadvantaged (67.8%), nurses displayed comparatively lower support (41.6%). This study did not attribute the differences to the personal characteristics of the two groups [31]. Rosen et al. conducted a nationwide survey involving 12,897 nursing, medical, and social work faculty and students in 47 nursing, 11 medical, and 15 social schools in the United States. The data revealed that social work professionals expressed the highest positivity towards abortion, while nursing professionals displayed the least favourability. Perhaps surprisingly, Catholic students in all three health professions expressed more supportive views toward abortion when compared to their respective faculty mentors and advisors. In general, health professionals were more likely to approve of abortion in situations where it was necessary to protect the pregnant person’s health [34]. Similarly, Such-Baer (1974) reported on the emotional reactions to abortion work among social workers, nurses, and physicians (*n*=42) in a teaching hospital in the United States; social workers were least discomforted by abortion, physicians showed slightly more discomfort, and nurses were most discomforted by abortion work. The differences in reactions by profession were significant at a *p* < 0.05 threshold. Factors potentially contributing to such reactions were listed as professional orientation, type of patient care, and contact with the fetus. Negative views on abortion showed correlations with religion, personal choice in doing abortion work, preferences on abortion laws, and the method of abortion [35]. Decades later, Ball gathered data from 51 counsellors, 42 social workers, and 78 nursing students, employing a questionnaire, a demographic instrument, and the Abortion Knowledge Scale, revealing that factors such as religion, age, gender, and race were not significantly associated with abortion attitudes among these professionals [37].

Some of the studies, however, noted that factors such as religion and religiosity [20,30] were significantly associated with social workers’ abortion attitudes. For example, Bird et al. discovered in the survey of 504 social work students that though 66.7% of students believed that religion offered little to no guidance in their daily lives, 22.8% disagreed with the idea that abortion should be legal in all circumstances, and 20.6% agreed that late-term abortions should be illegal in the U.S. [20]. In a classroom study conducted by Hancock with BSW students (*n* = 85), the majority of whom identified as evangelical or religiously conservative Christians, students with strong religious ideology conceptualized abortion as illegal, sinful, and found it difficult to differentiate between personal views and social work values. The students’ views regarding social justice and oppression were categorized into three themes: an ethic of conformity, an ethic of individualism, and an ethic of care [5]. Decades prior, survey findings by Hertel similarly indicated that religion was a crucial indicator with regard to abortion attitudes among both nurses and social workers in Tennessee [30], a study that also examined political affiliation in relationship to abortion attitudes. The findings revealed that both nurses and social workers who attended church less frequently, regardless of their political leanings (whether liberal or conservative), were more likely to support abortion. Liberals were more likely to approve of abortion, with 87% in favor, compared to 56% of their conservative-identifying counterparts [30].

Attitudinal research has also examined the emotional reactions of social work students and professionals to prenatal testing [32] and responding to family issues such as abortion [33]. In LaPan’s study in 634 social workers, it was noted that social workers tended to feel slightly more negative towards individuals who chose not to abort after learning about a “Down Syndrome” diagnosis, compared to those who had no prior knowledge. However, their empathy towards the clients overshadowed these negative feelings (overall aggravation average = 1.40; overall sympathy average = 5.86, and willingness to help average = 6.29) [32]. In Floyd et al.’s study of 147 undergraduate social work students, 41 students identified abortion as one of the most challenging family issues, rating it as the most challenging 24 times out of a list of 40 issues. The study emphasized the need for educators to develop strategies to better equip students in providing better social services to their clients [33]. A commentary by Benes presents narratives from both doctors and social workers working in abortion hospitals. Verbal expressions of the social workers in the study indicated that the work of abortion could be overwhelming, also describing their work as ‘exciting,’ ‘interesting,’ ‘beautiful,’ ‘upsetting,’ and ‘frustrating’ all at the same time [40].

#### Abortion stigma and barriers faced by social workers.

Seven studies examined stigma and barriers faced by social workers in their abortion care efforts [13,41–45]. This research also discussed the competencies of social workers in supporting people facing barriers in abortion access [13,45]. These studies were set in the U.S. [13,42,45,46], the U.K. [41], Canada [44], and Australia [43], and are summarized as follows.

In a reflective piece drawing from two and a half years of social work practice in abortion care, Haszeldine depicted the daunting challenges confronted by women navigating unplanned pregnancies. For a woman who is single, divorced, or widowed, and who may lack the financial or emotional backing of her parents or the father of the child, the decision to pursue an abortion was shown to be particularly arduous. The medical system may exacerbate the situation if doctors display unsympathetic attitudes and hinder access to timely appointments [41]. In Hayes and colleagues’ more recent study, three social work abortion counsellors based in Australia reflected on their work with women in the 18–24-week gestation period, emphasizing the sociopolitical judgments and stigma associated with accessing abortion. They also acknowledged the strength, resilience, and courage demonstrated by women seeking post-18-week abortion services [43]. Sperlich conducted a thematic analysis of 39 abortion narratives using trauma-informed social work framework, revealing the pervasive presence of stress in the narratives. The potential sources of stress were identified as legal restrictions, abortion stigma, encounters with anti-abortion protesters, and personal hardships. The study underscores the necessity for social work interventions tailored to alleviate stress throughout the process of seeking and obtaining an abortion [45]. In another recent study, Lands collected data from abortion-related sub-Reddits (online discussions and content focused on niche topics chosen by contributing participants) by scraping 250 posts made by users on the online platform. Some of the issues being discussed in the posts included help with abortion logistics, physical experiences to expect during abortion, conflicting emotions, and comfort that people felt sharing in the online space. The needs of abortion-seekers were further categorized into three categories: 1) informational needs; 2) emotional support needs; and 3) a need for a sense of community related to the abortion experience. Such needs were then described with regard to core competencies of social workers, suggesting that social workers should apply their social work practice acumen to also support individuals who have undergone abortions [13]. Highlighting physical barriers in accessing abortion care, Ely and colleagues examined how distance from abortion clinics and rural location affected the return rate for a second appointment under mandatory waiting period policies. Despite over 12% of counseling attendees not returning for the abortion procedure, individuals seeking abortion travelled an average of 50.53 miles, significantly higher than the national average of 10.79 miles travelled to seek abortion care. The study recommends that social workers understand state-specific reproductive health policies to address barriers through practice and policy efforts [46].

Bringing to light some of the deterrents that impact social workers’ engagement in providing abortion care, in a cross-sectional survey of 203 social workers, Bell identified greater religiosity, conservative beliefs, Republican (U.S. conservative party) voting, “pro-life” stances, and lack of participation in family planning training among social workers as key attributes that prevent them from engaging in abortion care work. Conversely, factors associated with reduced barriers included urban practice and workplace incentives to discuss family planning with clients [42]. In a Canadian, Ontario-based study, van Berkel discovered in interviews with abortion workers, which included social workers, that abortion work creates tensions due to several reasons: professional isolation from colleagues, security and safety issues, personal ambivalence and how workspaces keep abortion services undercover for security reasons and to protect themselves from losing funding opportunities. Workers often adopt concealment strategies despite the challenges they face in providing care, while also expressing personal gratification and pride in their work despite associated tension and risks [44].

#### Social work and reproductive justice.

Ten studies approached the topic of abortion in social work practice through the lens of reproductive justice [14,15,21,47–53]. Two of these studies discussed global perspectives: one focused on the Supreme Court’s reversal of the Roe v. Wade decision, gathering opinions from social workers in New Zealand, Ireland, and South Africa [52]. The other study explored the role of social workers as advocates for reproductive rights, using case studies from Australia, the Republic of Ireland, and New Zealand [53].

Studies highlighted the importance of incorporating a reproductive justice framework in social work practice and research. Elaborating on diverse legislative constraints put on contraception and abortion rights in the United States, Smith emphasizes the role of social workers in advocating for those whose rights are being compromised. This involvement can take various forms, such as identifying opportunities to influence policy, voicing their experiences gained as counsellors, teaching social work ethics to students, and publishing on the topic of reproductive justice [47]. Gómez et al. advocate for an intersectional approach in social work practice to address health inequities using the lens of reproductive justice, highlighting social workers’ pivotal role in challenging punitive pregnancy legislation and ensuring universal access to abortion care and substance use treatment. The article suggests multiple ways for engaging reproductive justice lenses in social work scholarship and practice, serving as a means to reinforce the field’s ongoing dedication to closing the health gap [21]. Similarly, Poehling et al. explore interdisciplinary solutions for social work’s grand challenges using reproductive justice framework. The principles of reproductive justice offer an interdisciplinary framework that facilitates social work students’ critical thinking, self-awareness, and self-regulation and prepares them for professional dialogue and ethical decision-making [50]. Hyatt et al. emphasize incorporating a reproductive justice lens, with a focus on access, resources, and focusing on the experiences of people with diverse identities in social work practice to address legislative attacks on abortion rights [14]. Lavalette et al. highlighted the opinion of social work academics from New Zealand, Ireland, and South Africa on the implications of the Dobbs v. Jackson Supreme Court decision overturning Roe v. Wade in 2022. This thought piece expresses their anger and disappointment regarding the end of constitutional protections for abortion in the United States. They wrote on the state of affairs of abortion rights in their respective countries, the advocacy roles that social workers can take in promoting reproductive justice, and how abortion is a fundamental human right [52].

Researchers have examined the social work literature base to evaluate the frequency by which reproductive justice has been discussed in relationship to social work. Liddell reviewed articles for the search term “reproductive justice” between 1994 and 2018 among the top 50 social work journals and identified only 10 articles, out of which only one focused on abortion. This article reported on the lack of a well-developed legacy of reproductive justice in social work [15]. Beddoe explored contemporary literature on reproductive rights in social work, reporting that there is ambivalence in social work literature when it comes to taking a progressive stance on abortion. The study urges social workers to use reproductive justice framework at the intersection of feminist perspectives to advocate for abortion rights [51]. Beddoe and colleagues called for enhanced visibility of a reproductive justice lens in social work education and practice, employing case studies of abortion rights movements from Australia, the Republic of Ireland, and New Zealand. The case studies revealed that social work professional organizations were silent on advancing abortion rights due to the influences of political environment. Conversely, it was individual social workers and grassroots, intersectional reproductive justice movements that actively engaged in advocacy efforts aimed at decriminalizing abortion [53].

Studies also focused on exploring social work students’ perspectives on reproductive justice topics, including abortion, with a call to promote discussion on the topic in social work curricula. Furio and co-authors examined 123 social work students’ perspectives on normative expectations related to childbearing. Analysis of 90 articles on the topic of reproductive justice resulted in a survey that listed 20 themes. The results of the survey found that stigma surrounding abortion services, the need for science-based sex education for youth, and the sense of marginalization faced by individuals opting not to reproduce were among the top concerns. The results showed that students of all genders had comparable levels of awareness regarding these expectations, suggesting that personal experiences were not indicators of awareness [48]. Younes et al. highlighted the need for overtly covering reproductive rights in the social work curriculum. This involves incorporating topics related to reproductive justice and reproductive health services, including abortion, training social work students towards advocacy for people seeking abortion care, and emphasizing the profession’s commitment to upholding human rights, including autonomy and self-determination [49].

#### Social work and ethical considerations regarding abortion.

Nine studies researched ethical considerations for social workers when encountering abortion in their practice [54–62]. Brieland recognized the limited availability of bioethics literature for social workers regarding selective abortion and contraception in the late 1970s. The article offered summaries of court decisions such as Roe v. Wade and Doe v. Bolton, which established the right to abortion, as well as cases like Mather v. Roe and Poelker v. Doe, which prohibited the use of federal funds for abortion. These summaries aimed to assist social workers in navigating emotionally charged counseling situations involving abortion and contraception [54]. During a similar timeframe, Sammons mentioned that selective abortion and amniocentesis raised ethical issues that could conflict with social work values. The study advised social workers to be well-informed and sensitive to the outcomes of these interventions, including confidentiality and privacy concerns surrounding genetic profiles, as well as procedures for obtaining informed consent. This research also suggested that social workers should play a key role in analyzing genetic procedures and policies [55]. In a more recent study, Reamer explored ethical challenges raised for social workers following the U.S. Supreme Court’s recent decision on the Dobbs v. Jackson case. The paper calls upon social workers to become knowledgeable about key ethics-based risk management protocols, the National Association of Social Workers (NASW) Code of Ethics, and roles of legal consultation and skilled documentation to protect both the client seeking abortion and themselves [56].

Studies discussed the intersection of religious beliefs and ethical considerations in social work abortion counseling. In an article by Smith-Osborne and colleagues, survey data collected from 294 social workers revealed that stronger religious and spiritual affiliations were associated with reduced support for both lesbian and gay rights legislation as well as abortion rights [58]. Following this, and amidst recent changes that prohibited several Title X sites (U.S.-based government-funded resources for low-income healthcare-seekers) to provide abortion referral and pregnancy options counseling, Hollenberger et al. suggested that social workers should prioritize ethical practices regardless of their religious beliefs, and should not guide clients through pregnancy options counseling themselves, rather making transparent referrals to other social workers and professionals who would be able to present the full range of available options to the client [57]. Streets similarly suggested that social workers should respond to clients’ beliefs when addressing sensitive issues like abortion over and above their own personal beliefs. Such an approach involves acknowledging clients’ ultimate truths, recognizing religion’s societal impact, and, if necessary, withdrawing or referring cases where a social worker’s religious views impede their duties [60].

Studies emphasized a need to recognize and protect the conscience rights of social workers when their religious beliefs prevent them from providing abortion services and address policy conflicts between the Catholic Church and the NASW. Adams responded to a NASW document on conscience clauses, asserting that recognizing the conscience rights of professionals is crucial for the moral integrity of both the practitioners and the profession. The article contends that the imposition of abortion service provision upon social workers, regardless of their conscientious objections, undermines the ethical integrity of the profession. This research posited that mandatory referral policies in the case of abortion disregards the conscience of social workers [59]. Similarly, Constable argued that the profession should support the idea of conscience protection of social workers. This research suggested that the profession should not take a one-sided ethical stance to silence some social workers’ consciences regarding abortion, an issue that may be contrary to the individual’s religious beliefs or moral convictions [61]. In a much earlier study, Dendinger et al. investigated the challenges faced by personnel in Catholic social service organizations grappling with conflicting policies from the Catholic Church and the NASW. The authors proposed addressing this issue by creating explicit agency-specific policy statements and provided a framework to guide the development of such policies [62].

#### Social work and abortion policy and advocacy.

Nine studies looked at abortion policy and advocacy in social work [38,63–70]. This research noted the significant role of social workers in advocating for abortion rights. In an editorial piece, Johnson explored the historical development of abortion policy, emphasizing how social workers contributed to shaping impactful social policy changes by posing essential questions about abortion and contraception [63]. McCoyd conducted a qualitative study on the decision-making process of women facing pregnancies affected by fetal anomaly, revealing the profound isolation they often experience amidst the polarized abortion discourse. This research emphasized the crucial role of social workers to assist women in comprehending their abortion journeys within the broader social framework and to advocate for policies that prioritize supporting women’s choices over perpetuating stigmatization [64]. Bernadi examined public opinion trends on abortion from 1975 to 2006 and found that 50–59% Americans supported abortion legality, 21–33% backed unconditional legality, and 55–72% favoured limits determined by gestational age or week of pregnancy. This paper further emphasized that considering the changing social climate around abortion rights, social workers should champion a more informed and progressive dialogue about abortion though legislative advocacy, reform initiatives via litigation, engagement in social action, and analysis of social policies [38].

Papers analysed the impact of restrictive abortion policies and role of social workers in advocating for the abortion rights. Ely’s analysis identified insurance limits, mandatory waiting periods, and state-scripted counseling as obstacles disproportionately affecting poor and rural women. The study calls upon social workers to advocate for abortion rights by engaging in policy practice as a profession and by emphasizing the profession’s responsibility to participate in social justice advocacy through educational programs [65]. Jackson analyzed the correlation between state policy restrictions and abortion rates from 1988 to 2000 across all 50 U.S. states encompassing parental consent laws, mandatory delays, insurance bans, Medicaid funding, and abortion provider numbers. The study found parental consent laws to be statistically significant in its association to abortion rate. This research highlighted the role of social workers in advocating for social justice, especially for young women, who face among the most restrictions to abortion access [66]. Freeman highlighted how economically disadvantaged individuals faced access barriers despite court rulings that legalized abortion [67]. The study noted that Medicaid restricted payments to in-hospital services, whereas the majority of early legal abortions were provided in outpatient clinics. This research then underscored social workers’ roles in addressing restrictive policies and reducing barriers to equitable abortion access [67]. Lieberman et al. made a similar argument that multiple federal and state legislative decisions limited government funding for abortion services and these restrictions impact young, rural, and poor women the most. The study urged social workers to make themselves available to counsel women with unplanned pregnancies [69]. Pollack examined discrimination against individuals with disabilities, highlighting legal complexities in their capacity for informed consent in abortion and sterilization cases. The paper then provided guidelines for social workers who were working with clients who have mental challenges, and suggested that social workers can strictly adhere to legal requirements, provide timely services, assess patients’ coping abilities, ensure understanding of medical procedures, obtain documentation from physicians, prevent coercion, prioritize patients’ welfare, allow consent withdrawal, understand surrogate consent laws, and uphold patients’ rights to autonomy and informed consent, while being familiar with state laws [68].

Mandelis investigated online resources on abortion, contraception, and parenting for Canadian internet users, revealing that pro-choice sites provide accurate information, while pro-life sites offer interactive but biased content. The analysis emphasizes the internet’s role in generating reproductive rights discourse in Canada. The author notes its direct influence on social work practice and policy, highlighting the significance of guiding clients to trustworthy online sources and engaging in advocacy for reproductive rights and public health education [70].

#### Social work and family planning roles, approaches, and practice frameworks.

Representing one of the most common themes discussed throughout the literature base on social work’s role in abortion care, 28 studies examined different facets of family planning within the field of social work.

Studies consistently emphasized the central role of social workers in various aspects of family planning, including abortion, and where their counseling makes a substantial contribution. Based on the analysis of 1,879 patients with “problem pregnancies” who received social services, Addelson [71] recommended the availability of social work counseling for women seeking abortion and a need to prioritize the psychological needs of clients during their abortion experience. Glatfelter explored the support needs of women who experienced perinatal loss (e.g., miscarriage or “spontanenous abortion”, stillbirth), highlighting the positive impact of compassionate care by nurses and social workers. The research underscored the significance of such support in enhancing satisfaction and potentially improving overall well-being for these parents [72]. Ullmann conducted a study in which group discussions led by social workers were conducted for women seeking abortion, revealing concerns such as procedural assurance, guilt, ambivalence toward motherhood, clinic personnel attitudes, and feelings of isolation. This research highlighted the critical role of social worker counseling in managing associated stress [73]. McCoyd stressed the significance of counseling provided by social workers in helping clients establish realistic expectations regarding pregnancy outcomes, acknowledging that pregnancy can have both positive and negative consequences. These recommendations stem from data gathered from women who underwent pregnancy termination due to fetal anomaly [74].

Based on interviews with 15 families in the 1980s, Furlong recommended that social workers provide counseling to families facing pregnancy termination due to fetal “defects”, spanning before, during, and after hospitalization for the termination. The study specifically focused on families with at least one other child and investigated their process of disclosing the news of the loss to their children [75]. McCoyd conducted focus groups with social workers followed by qualitative interviews with four physicians and 30 intensive interviews with women to delve into the decision-making process for women facing the termination of pregnancy due to fetal anomaly. The study revealed that women often develop mythical expectations rooted in denial regarding the possibility of fetal anomalies, harbor misconceptions about prenatal testing, and hold inaccurate expectations regarding the experience and duration of grief. Emphasizing the significance of provider awareness, the study offered suggestions for assisting women in coping with grief [76]. Fertel et al. underscored the pivotal role of perinatal social workers in offering preparedness, clear information, and guidance to parents navigating difficult pregnancy decisions, highlighting how social work counseling can mitigate the guilt and stigma associated with abortion choices [77].

Assessing U.S. data from abortion patients regarding contraception use, Murshid et al. affirmed the crucial role of social workers in providing counseling and guidance on contraceptive methods for informed decision-making in family planning [78]. Based on the study on women’s emotions surrounding abortion, Faria et al. recommended that social workers acknowledge the individuality of each woman’s situation when counseling on abortion. The study also advised integrating contraceptive planning into counseling sessions to prevent future unwanted pregnancies and reaching out to non-contraceptive users and those potentially in need of counseling [39].

In a study by Price on aftermath of reproductive loss (e.g., miscarriage or spontaneous abortion, stillbirth, induced abortion, ectopic pregnancy, and medical terminations of pregnancy), depression, anxiety, and psychological distress were found to be frequent outcomes experienced. This research emphasized the importance of discussing reproductive loss within social work practice to break silence for clients, suggesting strategies such as integrating these discussions into routine assessments, empowering clients to define their experiences, helping them to differentiate between adaptive and complicated responses to loss, and assisting them to confront that a similar event could occur again [79]. Hare and colleagues conducted a study of 162 women requesting termination of pregnancy and found that 52% of women seeking abortion required support from medical social workers. Over half the women interviewed required either clarification of options or assistance in attitude adjustment, indicating that increased involvement of medical social workers could significantly aid patients in their family planning decision-making process [80].

In the 1970s, Sung outlined the importance of social workers’ involvement in family planning for “indigent” mothers and children. The study emphasized collaboration with social work schools, regular evaluations, and early targeting of impoverished populations’ experiences of severe health or social problems. Challenges named included issues such as abortion, adoption, aggressive-destructive behaviour, anxiety, child abuse or neglect, childcare, discipline problems, emotional dysfunction, family planning issues, and others [81]. Strng emphasized that government-backed maternity and infant care initiatives aim to assist mothers and children from low-income households in the U.S., and social workers played a crucial role in intervening during financial crises or stressful situations faced by women, offering family counseling to pregnant unwed teenage girls’ families. The study underscored the effectiveness of integrating family planning with social work support [82].

Tucker discovered a preference for male children among Indian immigrants in the U.S., with reasons such as inheritance, financial concerns, and dowry issues associated with female children. The study examined the motivations behind sex-selective abortions within this community, emphasizing the importance of cultural education for social workers to comprehend these factors within the abortion context [83]. Fisher explored the topic of repeat abortions based on experience with clients in a London-based hospital. The study noted the complexity of counseling in such cases, illustrating the pivotal role of social workers. Fisher emphasized the importance of social workers going beyond societal perceptions regarding women who undergo multiple abortions. Instead, they should consider and respond to the potential underlying emotions of clients within their counseling approach [84].

Two studies examined the role of social workers in counseling adolescents before abortion. Cain discussed challenges faced by adolescents in need of abortion in the American context, including limited decision-making autonomy, familial fear, and societal reluctance to accept their sexuality. Social workers are tasked with providing neutral information, facilitating informed decision-making, and reassuring adolescents that abortion is just one aspect of their journey toward adulthood [85]. Similarly, Zakus et al. emphasized the susceptibility of adolescents to unwanted pregnancy and highlighted the important role that social workers can play through specialized counseling. This includes facilitating informed decision-making about abortion, assisting in coping with the procedure, and managing emotional challenges post-abortion. Additionally, social workers involved in abortion programs can contribute by providing valuable information within school settings [86].

Writing in the 1970s about the introduction of family planning to social work curricula, Hildebrand highlighted the absence of standardized training for abortion counsellors in the U.K., advocating for social workers to receive effective training in abortion counseling. The study suggested that post-qualification courses could lay the foundation for an innovative and impactful service to the clients in need of abortion counseling [87]. Haslett analyzed forty-two curriculum packages for early adolescents to explore the incorporation of family planning into social work education. The study found that the curricula had a moderate stance on gender roles and contraception but had a more conservative approach towards topics of premarital sexual activity and abortion. The study suggested that social workers, with their holistic approach and cross-cultural experience, are ideally positioned to lead the improvement of sex education curricula [88]. Hollenberger, in a teaching note, discussed that social workers can successfully support family planning needs of the clients like preconception care, pregnancy, abortion and adoption, and therefore it is essential to introduce family planning information in social work education by offering practical tools for integration into Bachelor of Social Work (BSW) curricula. Such curricula would also help them to address their personal biases and beliefs regarding issues of abortion [89].

Studies also highlighted various staffing and counseling models for social workers and other healthcare staff designed to improve abortion services. In 1970, and prior to the Roe v. Wade decision, the State of New York legalized abortion for any consenting woman less than 24 weeks pregnant, which led to high demand for abortion services in the hospitals. Looking at this update in the early 1970s, Mandel proposed a staffing model for first and second-trimester abortion services to minimize personnel cost and achieve a satisfactory level of quality patient care. The model explained staffing standards for physicians, anaesthesiologists, nurses, and social workers [90]. Similarly, Gameau described how the obstetric department at Queen Elizabeth Hospital in Australia developed a social work program to efficiently support high-risk patients seeking elective termination of pregnancy, aiming to improve services for the high numbers of abortions performed [91]. Hyatt proposed the ACCEPT MODEL for clinical social workers to use when counseling people who had terminated a pregnancy due to fetal anomaly. The model integrated a variety of grief theories and evidence-based interventions and offered a valuable framework for social workers to support clients in processing their experiences of loss [92].

Studies also discussed counseling approaches in the context of abortion. Surveys by Ely and colleagues examined the use of feminist social work practice philosophy in providing abortion counseling. The study revealed that most respondents were satisfied with the pre-abortion counseling aligned with feminist social work practice; 95.6 percent of the sample noted that their social work counselors provided accurate information. The study indicated that patients could benefit from social work counsellors who are knowledgeable about the philosophy of feminist counseling [93]. This body of research suggested that flexible and client-directed feminist social work counseling philosophies are valuable for guiding pre-abortion counseling sessions [89]. Layer assessed the effectiveness of a spiritual grief group in mitigating post-traumatic stress disorder (PTSD) symptoms among women experiencing post-abortion grief (PAG), revealing a notable decrease in shame (p < 0.001) and PTSD symptoms (p < 0.002) [94]. The study underscores the importance for social workers to screen for PAG in women and, when appropriate, refer them to agencies offering such supportive groups [94]. Ely employed a trauma-informed approach to investigate abortion-related hardships for patients in the U.S. who received financial pledges from the National Network of Abortion Funds’ (NNAF) Tiller Memorial Fund, to pay for an unaffordable abortion, revealing that participants encountered an average of 2.29 hardships, encompassing issues such as long travel distances for abortion, lack of birth control use, birth control failure, unemployment, and unstable housing [95]. The author suggested that social workers adopt a trauma-informed social work framework when engaging with clients during the abortion experience to mitigate and prevent trauma and trauma triggers [96]. In her exploration of feminist ethics of care, Suslovic compared her experiences working at a hospital with those at an abortion support collective. A stark contrast emerged in the comprehensive support offered by the city’s abortion support collective, where volunteers provided physical, logistical, emotional, and practical assistance throughout the abortion process. This study noted significant implications for feminist social work practice arising from the collective’s model [97].

## Discussion

Social work is uniquely positioned to have an important presence in abortion care, advocacy, and research. This review demonstrates that social workers are, and for decades have been, embedded in spaces to provide advocacy and support for those seeking abortion care [15,21,30,31,34,40,41,63,67,81,82,85,90]. Though this review covers a considerable 50-year timeframe, the literature base indicates that social workers have long been important members of the abortion care workforce, and that their work in this area is quite diversified. Providing medically accurate information, counseling patients and connecting them to resources, increasing access to abortion care, decreasing stigma among colleagues providing and people seeking abortions, and advocating for policy changes are among the range of care and support that social workers provide [7,9,43,44,47]. This is unsurprising as the profession is committed to the same social justice tenets that underpin abortion care and rights. Despite social work’s long-standing, if less recognized, presence in the abortion care space, gaps in education, training, and consciousness raising among social workers and in social work education persist. Addressing these gaps, and the coinciding stigma engulfing abortion care and rights, necessitates a reproductive justice approach to social work education, training, practice, policy advocacy, and research [14,15].

Existing research describes pathways for advocacy that include employing a reproductive justice lens in scholarship and practice [21] to address the myriad gaps in social work’s connections to universal abortion care, supportive pregnancy care, and health inequities [14,15,21]. Reproductive justice also offers a framework for increasing effective social work practice in abortion care and addressing gaps in the abortion care and social work research base [15,21]. Social work has made substantial contributions to improving family planning policies and access which positions social work for an increased and effective presence in abortion care practices and scholarship [15,73,80,82,87].

Given this history and the alignment of social work practice with reproductive and social justice tenets, there are myriad opportunities to increase the knowledge and practice bases of social work with regard to abortion care [38,46,62–70]. Social workers are embedded in organizations and at the intersections of medical, behavioural, community-based, and school health, providing a natural entry point for supporting abortion care [18,22,38,47,51,69,82]. Social work has established practice frameworks for reproductive health care. These include supporting family planning, accessing financial resources for low-income patients, and counseling on experiencing reproductive loss [76–79,82]. There are also practice frameworks for abortion counseling with pregnant adolescents and patients facing termination due to fetal anomalies [75]. These frameworks comprise a spectrum of abortion care support depending on patients’ medical and social needs, accessing abortion care, experiencing and the aftermath of pregnancy loss, and navigating the legal mandates [74,79,84,90]. Social workers have also played crucial roles in counseling contraception and providing sex education [85,88,98]. Research also suggests that patients who receive social work support during abortion care have positive experiences [89,93,94]. Building the research base and leveraging the professional experience of social workers in reproductive health offers a reservoir of resources for social workers and scholars to draw upon and to advocate for policy changes [14,15,21,47–53]. For example, social workers are also powerful policy advocates as they have hand-on and first-person anecdotal experiences counseling patients and navigating barriers to abortion care, making way for persuasive policy testimonies [47].

Social workers’ professional competencies are distinct from, and complementary to, the skills of healthcare providers, as empathy, communication, active listening, critical thinking, and advocacy are among the key skills that social workers emphasize in supporting those seeking reproductive health and/or abortion care [16,17]. The provision of non-judgmental and person-centered information, counseling, and connection to resources takes the whole person into consideration [16,17]. Social workers provide information, access to emotional, informational, and instrumental supports, and these connections are vital when supporting patients seeking abortion care [3,4]. Given that abortion care is highly stigmatized for patients and providers alike, the need for social workers to be well-trained in abortion care would support informed and compassionate decision-making, accessing medically accurate information, and increasing patients’ autonomy in making decisions about their families and healthcare [3,4].

Social workers adhere to a Code of Ethics that directly aligns with reproductive justice principles and underlines the key role of social work in abortion care [16,17]. The ethical principles of service, respecting autonomy, social justice, and dignity and worth of the person overlap significantly with reproductive justice principles of the right to have children, to not have children, and to parent children in safe and healthy environments [14,16,17]. Social work and reproductive justice principles are also underpinned by social justice and the intersectional phenomena that drive social inequities [14,15,21]. In addition to having responsibilities to clients and colleagues, for example, providing accurate information to clients, social workers have ethical responsibilities to the broader society [16,17]. These responsibilities include promotion of social welfare and social and political action [16,17]. The ethical responsibilities of social workers to broader society – promoting social welfare and social and political action - are similarly a core function of reproductive justice as a movement [14,16,17]. Reproductive justice as a movement and theoretical framework center the experiences of, and impacts of colonialism on, marginalized groups [14,15]. Social workers’ commitment to holistic treatment of the whole and autonomous person, are also core principles of reproductive justice [8–15,50]. This commitment naturally intersects with systems and individuals harmed by the structures (e.g., colonialism, structural racism, poverty, sexism) elucidated by the reproductive justice framework and scholarship [14,21,47,50–53]. The ethics and values are congruently embedded in social work practice and in reproductive justice advocacy, making both natural fits with each other [8–15,50].

The research base shows that while social workers align with and are well-placed to advocate for reproductive justice and abortion care, there are significant gaps in education, comprehensive social work professional policies and practice frameworks, and efforts to destigmatize abortion care among social workers [20,22–24,26,88]. Studies consistently show that social work students are not receiving adequate education and training on abortion care support or the legal contexts of abortion in their jurisdictions [23,25,26,36]. This is a significant gap as social workers are naturally embedded in social and health care organizations yet lack sufficient training or practice frameworks to support abortion care [2–4,6]. These education and practice gaps are compounded by the stigmatization of abortion [23,25,26,36,42]. There is a significant need to destigmatize abortion care and educate social workers and social work students about the continuum of reproductive justice issues that they may encounter in their careers [23,25,26,36,42]. Research indicating opposition to abortion rights among social work students is concerning and surfaces a need for consciousness raising and intersectional feminist approaches integrated into social work education and practice [5,20,24,30]. The absence of abortion care and rights discussed in social work education also supports a consciousness raising and intersectional feminist/reproductive justice integration of social work education, practice, and practice frameworks [48,49,73–75,78,80,82–84,87,90–92]. This may be particularly important in areas where abortion rights are significantly restricted, areas of high religiosity, areas where disinformation campaigns are focused, or for social workers working within religiously affiliated organizations [13,24,26,45,65–68].

This underscores a tension identified in the literature regarding negative attitudes toward abortion and conflicts with social work’s Code of Ethics [54–58,60]. Some studies identify efforts among social workers to maintain a “moral” objection to abortion and as well as their professional credentials, similar to pharmacists and physicians [57,59–61]. Ambiguity and debate do exist across the Codes of Ethics of medicine, pharmacy, and nursing [98,99]. In these professions, in which individuals choose to undertake education and/or training, the duty of care to the patient or supersedes the personal beliefs of the provider [98,100]. This duty is, or should be, upheld despite divergent religious beliefs between provider and patient or opinions of a person’s choices or life circumstances and specifically is upheld concerning standard medical practice [98]. Allowing for conscientious objections to supporting those seeking abortion, or other standard medical practice, ultimately centers the provider or social worker rather than the patient or client, contradicting the purpose of social work and duty to dignity and humanity of the client [98]. Noteworthy, research indicates that conscientious objection protections for providers delay or prevent patients from accessing abortion care which simultaneously violate a patient’s individual rights as well as the provider’s Code of Ethics to do no harm while centering the patient [99]. This would further dehumanize abortion-seekers and lend legitimization of this dehumanization by the social work profession.

In a similar vein, social work also has an established history of upholding its commitment to social justice and personal autonomy through policy advocacy [47,53]. This includes policy advocacy to influence laws supporting abortion rights and health equity, advocating for practice framework changes and improvements using an intersectional feminist lens, and establishing interdisciplinary frameworks or advocacy [14,21,50,52]. Such advocacy and interdisciplinary approaches also include expanding social work scholarship and public-facing dialogue related to reproductive justice [15,51,53]. Social workers may also mitigate the adverse effects of providers’ anti-choice attitudes toward abortion rights and care [31,34]. As social workers play critical and supportive roles in improving abortion care, policies, social work practice frameworks, interdisciplinary practice frameworks, and advocating for reproductive justice across these elements, there are many existing and natural points of entry for social workers to support abortion care. However, there are gaps in social work education and practice that need to be addressed. Additionally, systemic changes to better incorporate social workers in the continuum of reproductive health care would improve access and the experiences of receiving abortion care. The need for well-trained social workers in this space is increasingly important as the legal landscape of abortion is shifting along with the availability and rapid spread of misinformation about abortion [49,51]. Leveraging the long history of social work within abortion care and advocacy spaces will help increase access to accurate and safe abortion care. High quality and empathic education and training for students and professionals is needed to address gaps in knowledge, practice, and policy advocacy. This includes counseling practices, advocacy for patients and policies, and the destigmatization and/or consciousness raising about abortion rights across the profession. Reproductive justice offers a useful framework for integrating and advocating for abortion rights, autonomy, and centering marginalized communities, and the social justice tenets to which social work is committed [14,21,47,50–53].

### Strengths and limitations

Strengths of this review lie in its focus on the topic of abortion in social work, which is an area that has not been extensively synthesized with regard to its literature base. Additionally, the inclusion of peer-reviewed and grey literature, along with a wide timespan of studies from 1973, enhances the depth and breadth of the review. The article addresses a specific geographic region including countries including the United States, Canada, the United Kingdom, Australia, and New Zealand. This choice regarding study inclusion stems from a recognition that social work practices exhibit considerable variation across different geographical contexts but share relative similarities within these regions. While this focus may constrain a comprehensive understanding beyond these boundaries, it also ensures a degree of alignment with the topic. In addition to excluding other geographic areas, limitations highlight the overall lack of research on social workers’ engagement with more specific equity-deserving groups in connection to abortion information-seeking and navigation; this highlights an important gap that should be considered in future research. This review also underlines the evolution of language over time, as many studies used not only gendered, women-centric language, and most studies excluded discussions of the experiences of transgender, non-binary, and/or pregnancy-capable individuals, reflecting another significant gap in representation within the research. There were also studies included that use what is now considered antiquated, if not disparaging, language regarding fetal anomalies and other descriptors of both people and conditions impacting them. Of note, language documented in the studies pertains exclusively to the studies unto themselves and does not reflect the language of the authors. Though data were not extracted to specifically comment on how literature on social work and abortion ebbed and flowed in terms of topics covered in a temporal sense, it is evident that social work’s involvement in abortion care has remained both consistently present and diverse over the last half-century. Similar to other scoping reviews, this study is limited to the time bounds used for the searches. Research that has emerged since the review was conducted would be excluded from the analyses.

## Conclusion

The primary aim of this scoping review was to know how abortion care has been discussed in the discipline of social work. The research identified various dimensions of social work’s involvement with abortion, including attitudes toward abortion and abortion-seekers, abortion stigma and barriers faced by social workers, the intersection of social work with reproductive justice, abortion policy and advocacy, family planning, and ethical considerations surrounding abortion. In conclusion, the scoping review summarizes the multifaceted role of social work in abortion, from providing client-centered support to advocating for policy changes that uphold reproductive rights and justice. Moving forward, it is imperative for social work education and practice to actively integrate reproductive justice principles, address abortion stigma, and navigate ethical complexities to ensure equitable access to abortion care for all individuals.

## Supporting information

S1 Table Studies included in review. (DOCX)

S1 Appendix Search term and strategy examplar. Keyword search strategy used, illustrating APA PsychInfo (Ovid) search terms.
(DOCX)
