# Supplementary material for: Understanding social work’s role in abortion care: A comprehensive scoping review
Source: PLoS One. 2025 Apr 24;20(4):e0320260. doi: 10.1371/journal.pone.0320260 (PMC12021202; doi:10.1371/journal.pone.0320260)
Supplement: S1 Table — (DOCX) [file pone.0320260.s001.docx]

**S1 Table.** Studies included in review.

| Author | Country | Area | Title | Aims/Objective | Methods | Relevant Findings/Results | Usefulness to Review |
| --- | --- | --- | --- | --- | --- | --- | --- |
| Adams P.  (2011) | USA | Practice | Coercing conscience: Professional duty or moral integrity | Reply to a National Association of Social Workers document about conscience clauses. | Conceptual/Thought Piece | Author argues that respecting the conscience rights of professionals is important for the moral integrity of both, practitioners concerned and of the profession itself. | Discusses conscience rights of social workers in abortion care. |
| Addelson F.  (1973) | USA | Practice | Induced abortion: Source of guilt or growth? | To describe the role of the social worker in the medical management of women requesting abortion in a hospital setting based on the experiences of patients. | Clinical Chart Review | Recommended the availability of social work counseling for women seeking abortion and a need to prioritize the psychological needs of clients during their abortion experience. | Set in 1973, the paper underscores the crucial role of social workers within the medical team in supporting abortion care. |
| Ball MJ.  (2010) | USA | Research | The abortion attitudes of counselor, social work, and nursing trainees | To examine the similarities and dissimilarities in abortion attitudes among counselors, social workers, and nurses in training, based on profession, age, race, gender, and religion. | Quantitative | The results indicated that religion, age, gender, and race do not significantly predict abortion attitudes in counselors, social workers, and nurses. The majority of the counselors, social workers, and nurses were "pro-choice," as measured by self-report. | Describes similarities and dissimilarities in abortion attitudes amongst different health professionals including counselors, social workers, and nurses. |
| Beddoe et al.  (2020) | Australia, the Republic of Ireland and New Zealand | Policy | "Social justice for all!" The relative silence of social work in abortion rights advocacy | To make a case for the increased visibility of reproductive justice within education and professional activity, employing case studies from various countries to illustrate recent social work advocacy on abortion rights. | Conceptual/Thought Piece | The social work abortion activists report two themes: professional bodies have varied their approach to advocacy for abortion rights due to political sensitivities; and social work involvement in campaigns has reflected individual and grass-roots advocacy. | Explores the significance of integrating a reproductive justice perspective into both social work practice and education. |
| Beddoe L.  (2022) | New Zealand | Policy | Reproductive justice, abortion rights and social work | To explore contemporary literature on reproductive rights, in particular, abortion rights, and social work. | Conceptual/Thought Piece | This piece reports that there is ambivalence in social work literature when it comes to taking a progressive stance on abortion. The study urges social workers to use reproductive justice framework at the intersection of feminist perspective to advocate for abortion rights. | Speaks about involving more conversation on reproductive rights, including abortion, using intersectional feminist lens in social work. |
| Begun et al.  (2016) | USA | Education | Correlates of social work students' abortion knowledge and attitudes: Implications for education and research | Examine the relationship between social work students' pre-existing knowledge about abortion and their attitudes towards abortion. | Quantitative | 81.5% of participants indicated limited classroom discussions on abortion, with 57.5% indicating confidence in assisting clients seeking abortion.  22.8% students disagreed with the idea that abortion should be legal under all circumstances, and 20.6% students agreed that late-term abortions should be illegal. | Emphasizes the importance of integrating accurate reproductive health content, including abortion, into social work education. |
| Begun et al.  (2017) | USA | Education | Exploring U.S. social work students' sexual attitudes and abortion viewpoints | Do social work students' attitudes toward sexual permissiveness correlate with their views on abortion? Are there associations between social work students' attitudes toward birth control and their opinions on abortion? | Quantitative | Linear regressions indicated that those endorsing permissive sexual attitudes and supportive attitudes toward birth control were less likely (β = −.43, p < .001) to hold anti-choice abortion views. | The study suggests enhancing social work education regarding sensitive reproductive and sexual health topics by emphasizing diversity, human rights principles, and professional ethics alongside the subject matter. |
| Bell et al.  (2008) | USA | Research | Barriers in the provision of family planning information from social workers to their clients | To study the barriers that social workers face in providing family planning information to their clients. | Quantitative | Religiosity, conservative politics, Republican policy-makers, and a "pro-life" stance heightened obstacles to family planning information, whereas family planning coursework and urban practice lessened barriers.  Respondents with greater religiosity reported a higher barrier level in providing family planning information (p<.0001)   Respondents who identified as “pro-choice” regarding abortion rights reported fewer barriers in providing family planning information than those who do not identify as “pro-choice” in respect to abortion rights (p<.0001). | Describes barriers faced by social workers in family planning including abortion. |
| Benes M.  (1976) | USA | Practice | Performing abortions | To understand doctors' and social workers' perspectives as they provide abortion related services. | Commentary | Author shares observations from her visit at the abortion clinic. Narratives of social workers express the range of emotional reactions they feel during the abortion procedure. | Provides insights in emotions of the professionals providing abortion services. |
| Bernadi et al.  (2012) | USA | Policy | Abortion, partial-birth abortion, and adolescent access to abortion: An overview for social workers | To critically examine public opinion trends in the arenas of abortion, partial-birth abortion, and adolescents’ rights to access abortion in the context of social work practice. | Quantitative | From 1975 to 2006, American attitudes on abortion were steady: 50-59% supported legality, 21-33% backed unconditional legality, and 55-72% favored a partial-birth abortion ban. Consent for abortions among women under 18 fluctuated between 69-74%. | Discussed importance of social workers promoting an informed and progressive discourse on abortion, given shifting societal attitudes towards abortion rights. |
| Bird et al.   (2018) | USA | Practice | Religiosity and personal beliefs regarding abortion: Results from a survey of social work students in the United States | To explore potential associations between religiosity and abortion attitudes in the social  work profession. | Quantitative | Most students (66.7%) note they lack religious guidance in daily life, hold varied views on abortion, and a notable percentage (27.8%) expressed confidence that they would make better reproductive health decisions for their clients. | Discusses the crucial role of social workers in supporting clients' autonomous decision-making on abortion, irrespective of the social workers' personal beliefs. |
| Brieland D.  (1979) | USA | Policy | Bioethical issues in family planning | Review of court-established policies on contraception and elective abortion for social workers. | Policy Analysis | Summarizes court decisions on the legal aspects of contraception, selective abortion, and abortion, aiming to assist social workers in understanding key family planning issues. | Provides legal summaries of abortion cases to help social workers as they deal with emotion-laden issues that make counseling difficult. |
| Cain L.  (1979) | USA | Practice | Social workers' role in teenage abortions | To understand the role of counseling by the social worker when interviewing an adolescent prior to an abortion. | Clinical Chart Review | The social worker plays a crucial role in balancing a teenager's autonomy and potential family involvement, facilitating discussions about options such as abortion or adoption. | Explains importance of counseling by social worker for an adolescent or teen seeking abortion. |
| Constable R.  (2013) | USA | Policy | Social workers, conscience protection and practice | To review conscience protection law and ethics in social work from different perspectives. | Conceptual/Thought Piece | Social work profession should support the idea of conscience protection of social workers. The social workers should have the right to not get involved in cases that are against their religious beliefs or moral convictions such as abortion. | Reflects on the significance of conscience protection law in social work profession when dealing with a client seeking abortion. |
| Dendinger et al.  (1980) | USA | Policy | Abortion: Toward developing a policy in a catholic social service agency | Explore counseling services for problem pregnancies and abortion in Catholic social agencies, assessing conflicts between the Roman Catholic Church's policies and the National Conference of Catholic Charities. Consider how social workers navigate these conflicts, in alignment with the National Association of Social Workers and professional Code of Ethics. | Conceptual/Thought Piece | This article discusses the conflict personnel in Catholic social service organizations face regarding abortion policies. The authors recommend creating explicit policy statements within each agency, offering a suggested policy to assist in navigating the issue. | Reflects on abortion issues within the framework of Catholic social service organizations and the social work profession. |
| Ely et al.  (2019) | USA | Research | Access to abortion services in Tennessee: Does distance traveled and geographic location influence return for a second appointment as required by the mandatory waiting period policy? | Analyze the distance traveled, return rates, and differences between rural and urban zip codes for accessing abortion procedures during a 48-hour mandatory waiting period in Tennessee. | Quantitative | Despite over 12% of counseling attendees not returning for the abortion procedure, individuals seeking abortion travelled an average of 50.53 miles, significantly higher than the national average of 10.79 miles. The study recommends that social workers understand state-specific reproductive health policies to address barriers through practice and policy efforts | Explains the role social workers can play in promoting social justice and helping people better access abortion services. |
| Ely et al.  (2010) | USA | Policy | Abortion policy and vulnerable women in the United States: A call for social work policy practice | Examine abortion policy, specifically insurance coverage, Medicaid restrictions, mandatory waiting periods, and state-mandated counseling. Explore the advocacy role of social workers in shaping and influencing abortion policy. | Conceptual/Thought Piece | Insurance limits hinder abortion access for health reasons. Mandatory waiting periods disproportionately affect poor and rural women, questioning their decision-making. State-scripted counseling aims to undermine women's confidence in moral choices. The study urges social workers to advocate for women's abortion rights through policy engagement and educational programs on social justice. | Article talks of the urgent call for social workers to lead in addressing abortion access issues for vulnerable women. |
| Ely et al.  (2010) | USA | Research | An examination of levels of patient satisfaction with their abortion counseling experience: A social work practice evaluation | Investigate patient satisfaction with pre-abortion counseling sessions within the framework of feminist social work practice philosophy. | Quantitative | The majority of patients who responded to the survey were satisfied with their counseling experience. 96.2% of participants reported that counseling session was helpful. | The paper details feminist social work counseling with regard to abortion decision-making. |
| Ely et al.  (2012) | USA | Practice | Social work student attitudes toward the social work perspective on abortion | To examine social work student attitudes towards the social work profession’s broader perspective on abortion in the United States. | Quantitative | 49% of students perceive that they would not make a referral for abortion and 41% did not know whether or not abortion was legal in their state, and higher religiosity showed significant associations with reduced acceptance of abortion, as well as lower likelihoods of making abortion referrals. | Helps understand how factors like religion may impact practice of social work professionals in abortion counseling. |
| Ely et al.  (2017) | USA | Research | A trauma-informed examination of the hardships experienced by abortion fund patients in the United States | To use a trauma-informed lens to explore abortion-related hardships for patients in the United States who received financial pledges from the National Network of Abortion Funds’ (NNAF) Tiller Memorial Fund, to pay for an unaffordable abortion. | Quantitative | On average, patients experienced 2.29 hardships accessing abortion. Most frequent hardships included having multiple children (37.8%), currently on public assistance (37.1%), traveling over 50 miles for an abortion (28.8%), not using birth control (24.2%), birth control failure (22.3%), unemployment (19.6%), and unstable housing (18.7%). | Explains use of trauma-informed care framework by social workers in abortion care. |
| Ely et al.  (2018) | USA | Research | A trauma-informed social work framework for the abortion seeking experience | Examine the abortion-seeking experience concerning stress, trauma, and trauma-informed care. Highlights the advantages of adopting a trauma-informed care approach and propose a model applicable to the abortion-seeking experience in the United States. | Conceptual/Thought Piece | Potential stressors during the abortion-seeking process include unintended pregnancy, policy barriers, provider access, financial insecurity, clinic environment, and abortion stigma. Social workers should employ a trauma-informed care framework to mitigate stressors for these patients. | Explains use of trauma-informed care framework by social workers in abortion care. |
| Faria et al.  (1985) | USA | Research | Women and abortion: Attitudes, social networks, decision-making | To investigate women's attitudes and emotions regarding abortion, explores the sources of support they seek when deciding to undergo the procedure, and examines the specific reasons influencing their choice. | Quantitative | 72% women sought guidance on pregnancy decisions. Women saw abortion positively for unwanted pregnancies; Protestants favored abortion more strongly than non-Protestants.  The study recommended that social workers acknowledge the individuality of each woman's situation, integrate contraceptive planning into counseling sessions and reach out to non-contraceptive users and those potentially in need of counseling. | Study describes attitudes of women seeking abortion and how social workers can help them based on the findings pertaining to each group under study. |
| Fertel et al.  (1997) | USA | Practice | Counseling prenatal diagnosis patients: The role of the social worker | To explore counseling techniques and therapeutic strategies that can be employed by perinatal social workers when assisting parents facing difficult decisions related to their pregnancies due to negative prenatal diagnosis. | Clinical Case Example | Social workers can prepare by reviewing charts and consulting with healthcare professionals, ensuring effective interviews. Clear written information can be provided to help patients absorb details at their own pace, and information about procedures and resources can also be shared. | Highlight important role of social workers in counseling families who face difficulty making pregnancy decisions including abortion. |
| Fisher S.  (1986) | UK | Research | Reflections on repeated abortions: The meanings and motivations | The paper explores the factors related to repeated abortions based on the writers’ experience with clients in the hospital. | Conceptual/Thought Piece | Study speculated that for certain women, abortion could serve as a transition from one self-perception to another, or from dependency to independence. It's possible that the decision to have an abortion might unconsciously aim to address or heal early developmental setbacks, leading to a satisfactory resolution. | Reflects on repeat abortions and significance of abortion counseling by social workers in these cases. |
| Floyd et al.  (2011) | USA | Practice | Baccalaureate student perceptions of challenging family problems: Building bridges to acceptance | To explore how BSW students respond to difficult family issues like abortion, addiction, and LGBTQ+ matters, categorizing their reactions as emotional, cognitive, or experiential. | Quantitative | 41 students identified abortion as one of the most challenging family issues to address. Among a list of 40 issues, abortion was rated as the most challenging 24 times. The thematic qualities of the shared reactions were categorized into affective, cognitive, or experiential. | Offers a framework for instructors to recognize and address barriers encountered by students when dealing with challenging family issues. |
| Freeman EW.  (1976) | USA | Policy | Abortion: Beyond rhetoric to access | Highlight bias in accessing legalized abortion for economically disadvantaged individuals and outline social workers' role in addressing policies limiting equitable access. | Conceptual/Thought Piece | Healthcare discriminates, offering sterilization to poor individuals seeking abortion. Medicaid is restricted to in-hospital services only. "Medically necessary treatment" is read subjectively. Some professionals show negativity, and social work overlooks abortion's complex aspects. | Underscores the vital role of social workers in advocating for abortion services and highlights their efforts in delivering safe, confidential, and efficient care to those in need. |
| Furio et al.  (2022) | USA | Research | Normative expectations on childbearing: A study of reproductive justice | To facilitate a deeper discussion on reproductive justice in the field of social work and to examine social work students' perspectives on normative expectations related to childbearing and reproduction. | Quantitative | Three of the top concerns expressed by students were: “Women who have utilized abortion services often feel shameful and are secretive about their decision”; “Women who seek abortion services are often stigmatized and marginalized”; and “There are women who have had abortions and still wish to parent in the future”. | Helps understands social work students’ perceptions about abortion stigma. |
| Furlong et al.  (1984) | USA | Research | Pregnancy termination for genetic indications: The impact on families | This paper explores how families who terminated pregnancies due to serious defects coped with the decision, termination process, and its impact on their families and children. | Qualitative | Parents faced a challenging decision-making process during termination. Children received varying explanations of the loss, with emotions documented. Recommends social workers to offer valuable support to assist patients and their families before, during, and after hospitalization for the termination. | Highlights the family crisis during abortion and role of social work counseling. |
| Gameau B.  (1993) | Australia | Practice | Termination of pregnancy: Development of a high-risk screening and counseling program | This paper describes the development and implementation of The Queen Elizabeth Hospital social work program for public patients who come for elective termination of pregnancy for psychosocial reasons. | Program Development | The paper explains the elements of social service program for abortion counseling including social work screening and referral that incorporated high-risk indicators and a pre-abortion counseling protocol. | Elucidates the process of designing programs tailored for social workers, enabling them to effectively support high-risk clients in navigating their pregnancy decision-making processes. |
| Glatfelter KS.  (2017) | USA | Research | Understanding and addressing the needs of women experiencing perinatal loss leading to hospital protocol change | To examine what women need to feel supported, emotionally, physically and spiritually within a hospital setting and how these supports impact women experiencing perinatal loss. | Qualitative | Compassionate care by nurses and social workers improves women's satisfaction and potentially their health. Professional, sensitive support enhances women's experiences, while those without personal experience of perinatal loss may lack the understanding needed to provide meaningful help. | Discusses interventions to assist social workers to support the women experiencing the loss. |
| Goldblatt Hyatt ED.  (2021) | USA | Practice | Counseling women who have terminated a pregnancy due to fetal anomaly (TOPFA): The ACCEPT model | To provide a practice model through a composite  case study utilizing ACCEPT model for an informed approach to therapeutic intervention for clinical social workers towards women who have terminated a pregnancy due to fetal anomaly. | Practice Model Development | ACCEPT MODEL integrates a variety of grief theories and evidence-based interventions. This model has following stages: -  1. Beginning Phase: Stabilization: Acknowledging  the Loss and Connecting Emotions  2. Middle Phase: Continuing Bonds, Exploring  Distortions, and Practicing New Skills  3. Ending Phase: Telling the Story | Describes concrete model that can be used by social workers to provide counseling to grieving clients and help them process their loss. |
| Gomez et al.  (2020) | USA | Policy | Advancing reproductive justice to close the health gap: A call to action for social work | To present an intersectional  perspective on health inequities through the lens of reproductive justice with an aim to bolster social work’s ability to close the health gap and foster health equity for all people. | Conceptual/Thought Piece | The reproductive justice framework can guide social work practice and academic research to actively support reproductive freedom. Social workers play a crucial role in advocating for the reversal of legislation criminalizing pregnancy and ensuring accessible abortion care and substance use treatment for all clients can further use RJ framework to close health gaps. | Articulate strong potential that social workers hold to advocate for reproductive rights through their practice. |
| Hancock TU.  (2008) | USA | Practice | Doing justice: A typology of helping attitudes toward sexual groups | To create a typology (categorization of attitudes) that categorizes the students’ views on justice and oppression and willingness to work in practice situations with which the students disagree with an issue on moral grounds. | Typology Development | Religious ideology, rural context and moral grounds can impact social work practice by students when dealing with human rights issues like abortion.   Author provided three typologies -an ethic of conformity; an ethic of individualism; and an ethic of care. Each orientation was named to reflect the theme that characterized the students’ social justice and human rights concerns regarding sexual groups. | Helps to understand separation of the “personal” from the “professional” in the area of social work in relation to abortion. |
| Hare et al.  (1981) | UK | Practice | Counselling needs of women seeking abortions | To assess the counseling needs of a group of women requesting termination of pregnancy. | Quantitative | Among 162 women, 8% needed medical advice primarily for social issues, while 52% sought assistance from the medical social worker for counseling and support. The findings emphasize the importance of counseling for women before and after abortion to aid decision-making. | Highlights the crucial role of social worker counseling for women throughout the abortion decision-making process. |
| Haslett D.  (1991) | USA | Research | Sex education curricula for young teens: Implications for social work | Assess sex education curricula for young adolescents, examining content and its relation to key variables (gender roles, premarital sex, contraception, and abortion) and curriculum sources. | Curriculum Review | Curricula adopted a moderate stance on gender roles and contraception but were restrictive on premarital sexual activity and abortion.  Social workers, with their holistic approach and cross-cultural experience, are ideally positioned to lead the improvement of sex education curricula | Contributes to understanding how social work curriculum talks about abortion and requirements for a change in the curricula to introduce topics like abortion. |
| Haszeldine V.  (1974) | UK | Practice | Abortion: A medical social worker's point of view | 1. Analyze patients' personal and medical experiences while seeking abortion.   2. Understand critical role of the hospital staff in working with abortion patients. | Conceptual/Thought Piece | This paper talks about experiences of individuals seeking abortion from a lens of a social worker. This includes emotions, life situations, decision-making processes, barriers, dilemmas, and anxieties that some face in their journey. | Presents the journey of people seeking abortion and reiterates the role of medical staff, including social workers, to be considerate to the needs and situation of the patient. |
| Hayes et al.  (2020) | Australia | Research | Counselling "late women"- The experience of women seeking abortion in the eighteen to twenty-four-week gestational period: Critical reflections from three abortion counsellors | 1. Compare counseling for women in the 18-24 week stage of pregnancy to those earlier in pregnancy/in first trimester.  2. Identify counseling frameworks and techniques for working with women in the second trimester.  3. Address public health and advocacy issues discussed by women in the second trimester for improved care. | Qualitative | Women in 18-24 gestational age range of pregnancy encounter judgment and stigma in accessing healthcare. Women show strength in seeking post-18 week abortions. Women navigate 'deserving' versus 'undeserving' narratives with resilience. | Discusses the importance of social workers providing accessible public counseling and support services for women in need of abortion. |
| Hendershot et al.   (1974) | USA | Practice | Abortion attitudes among nurses and social workers | Assess abortion attitudes in nurses and social workers. | Quantitative | Social workers favor abortion services for the economically disadvantaged (67.8%), but nurses demonstrated less support (41.6%), potentially causing issues in service delivery. | Emphasizes the positive influence of progressive training for social workers on abortion care. |
| Hertel et al.  (1974) | USA | Research | Religion and attitudes toward abortion: A study of nurses and social workers | To analyze rates of approval of abortion by liberal and conservative Christian nurses and social workers in Tennessee. | Quantitative | Religion shapes abortion views among nurses and social workers, with conservatives more likely to oppose abortion (56%). Frequent church attendance is linked to abortion disapproval for both conservatives and liberals. | Highlights relationship between religious and abortion attitudes. |
| Hildebrand J.  (1977) | UK | Practice | Abortion: With particular reference to the developing role of counselling | To discuss some of the existing problems in the area of abortion counseling for workers and clients alike. | Conceptual/Thought Piece | Social workers must be trained to counsel clients effectively, understanding the significant changes and anxiety associated with pregnancy.   Post-qualification courses could lay the foundation for an innovative and impactful service to the clients seeking abortion counselling. | Highlights training around abortion counseling for social workers. |
| Hollenberger et al.  (2021) | USA | Policy | Pregnancy options counseling, Title X, and social work: What does faith have to do with it? | To explore Family Planning, Pregnancy options counseling, then-emerging Title X regulations and their intersection with the evangelical Christian faith and social work. | Conceptual/Thought Piece | The study recommended that if social workers' values align with Title X changes, they should prioritize ethical practices, regardless of religious beliefs. Alternatively, they should refrain from counseling on pregnancy options and honestly refer clients to another professional for support. | Emphasizes ethical practices for social workers in abortion referrals, emphasizing the importance of setting aside personal religious beliefs. |
| Hollenberger et al.  (2021) | USA | Education | Integration of family planning information in social work education | To introduce family planning services and their significance to social work practice. | Conceptual/Thought Piece | Offers a fundamental overview of family planning, encompassing abortion and relevant literature. It concludes by providing practical tools for integrating these concepts into BSW curricula. | Addresses the need to add family planning topics to BSW curricula. |
| Hyatt et al.  (2022) | USA | Policy | From abortion rights to reproductive justice: A call to action | To call upon social workers to respond to the systemic and ideological injustices found in reproductive health care. | Conceptual/Thought Piece | emphasize incorporating a reproductive lens in social work practice to address legislative attacks on abortion rights and all other injustices done to reproductive rights of people.  Following overarching pillars of Reproductive Justice were discussed (a) the right not to have a child; (b) the right to have a child; and (c) the right to parent children in safe and healthy environments | Explains ways by which social workers can advancing reproductive rights by applying framework of reproductive justice to their practice and advocacy. |
| Jackson DL.  (2007) | USA | Policy | State policy restrictions on abortion: Implications for social workers | To understand the relationship in states between policy restrictions that impact access to abortion and states’ rate of abortion. | Quantitative | Five state-level correlations were observed: parental consent laws correlated with mandatory delays and insurance bans; Medicaid funding and abortion provider numbers showed a correlation; and a strong correlation existed between abortion providers and the proportion of minority women, possibly due to their role as control variables. | Helps to understand variables that contribute to changes in the relationship between state policies and the abortion rate. |
| Johnson BS.  (1978) | USA | Policy | Abortion: Yesterday, today, and tomorrow | Study historical development of abortion policy and the role of social workers in advocating for abortion as a public policy. | Editorial | The editorial underscores social workers' vital role in advocating for abortion rights, urging them to ask crucial questions about abortion and contraception for impactful social policy change. It prompts reflection on social workers' awareness of their role in shaping abortion policy. | Illustrates the depiction of social work and its importance in advocating for abortion rights. |
| Lands et al.  (2023) | USA | Research | "Am I the Only One Who Feels Like This?": Needs expressed online by abortion seekers | A qualitative analysis of a comprehensive, anonymous dataset from Reddit focused on capturing individuals' needs and emotions related to their abortion experiences. | Qualitative | Three interconnected needs of people seeking abortion emerged: (1) need for information, (2) need for emotional support, and (3) need for community around the abortion experience.   These needs align with the core competencies of social workers and therefore, social workers should apply their existing abilities to support individuals who have undergone abortions | Shows how social media engagement by social workers can inform social work practice in the area of abortion. |
| LaPan et al.  (2006) | USA | Research | Prenatal testing, birth outcomes, and views of social workers | To examine whether social workers have more negative views towards families knowingly choosing to have a child with Down syndrome. | Quantitative | Social workers tended to feel slightly more negative towards individuals who chose not to abort after learning about a Down syndrome diagnosis, compared to those who had no prior knowledge. However, their empathy towards the clients overshadowed these negative feelings (overall aggravation average = 1.40 and overall sympathy average = 5.86) and a willingness to assist the client (willingness to help average =  6.29). | Helps in evaluating biases of social workers while providing genetic counseling that might impact abortion outcomes. |
| Lavalette et al.  (2022) | Multiple countries- New Zealand, Ireland, South Africa, USA | Policy | Abortion rights and Roe v Wade: Implications for social work - voices from the social work academy | Three social work writers from across the globe write about abortion rights and the significance of the Supreme Court decision to overturn the historic ‘Roe v. Wade’ ruling, which made abortion legal in the U.S. as of 1973. | Conceptual/Thought Piece | Authors discuss the implications of reversing abortion rights. They have emphasized the fact that abortion is a fundamental right, there is a need to write social work action and suggest what social workers can do in their efforts to encounter challenges that this judicial decision brings for them in service. They also wrote on the state of affairs of abortion rights in their native countries. | Social workers speak in response to discriminatory abortion laws. |
| Layer et al.  (2004) | USA | Research | Postabortion grief: Evaluating the possible efficacy of a spiritual group intervention | Assess the efficacy of a spiritual grief group in alleviating PTSD symptoms among women with Postabortion Grief. Identify both beneficial and less effective aspects of the intervention for women undergoing Postabortion Grief. | Mixed Methods | Following the intervention, there was a significant reduction in shame (p < .000) and PTSD symptoms (p < .002). Over 80% emphasized the strong impact of their religious beliefs and the spiritual intervention in the group. | Underscores the importance for social workers to screen for postabortion grief in women grieving an abortion. |
| Liddell JL.  (2019) | USA | Research | Reproductive justice and the social work profession: Common grounds and current trends | To identify and review the current state of reproductive justice literature in the social work literature between 1994 and 2018 among the top 50 social work journals. | Literature Review | Only 10 articles identified, out of which only one focused on abortion. The articles collectively urge increased research on reproductive justice topics. While the author notes a lack of a well-established legacy of reproductive justice in social work, there are indications that some social workers are receptive to this approach. | Help us identify how reproductive justice has been addressed in social work literature. |
| Lieberman et al.  (1992) | USA | Policy | The role of social work in the defense of reproductive rights | Explore and analyze barriers to abortion, focusing on issues related to funding and access. | Conceptual/Thought Piece | Restrictions disproportionately affect marginalized women, especially those in poverty, rural areas, and with substance use. Unwanted children may face enduring social, psychological, and economic challenges, which often extend into their twenties and beyond. | Describes barriers to abortion services and impact on denial of abortion on women and role of social workers to carry out the ethical mandates of the profession. |
| Mandel MD.  (1974) | USA | Practice | An operational and planning staffing model for first and second trimester abortion services | To provide an abortion service staffing model for first and second trimester abortion services that helps to minimize personnel cost, achieve a satisfactory level of quality patient care, and conforms to the New York City Health Code. | Program Development | Model describes first and second trimester staffing standards for financial assessment, comprehensive medical screening, specified timeframes for staff involvement, and details on laboratory test time and cost. | Details staffing models to optimize healthcare system for providing abortion services. |
| Mandelis A.  (2013) | Canada | Research | Wealth and stealth: The 21st century challenge to comprehensive reproductive health education and services in Canada | Examine online resources on abortion, contraception, and parenting accessible to Canadian internet users. | Qualitative | Pro-choice sites provide accurate, static information, while pro-life sites offer interactive but biased content. The analysis underscores the internet's impact on shaping reproductive rights discourse in Canada, noting its direct influence on social work practice and policy. It stresses the importance of directing clients to reliable online sources and advocating for reproductive rights and public health education. | Comparison of pro-life and pro-choice websites with a social work lens. |
| McCoyd JL.  (2003) | USA | Research | Pregnancy interrupted: Non-normative loss of a desired pregnancy after termination for fetal anomaly | To explore how women, with little normative guidance, experience and make sense of their pregnancy loss within the framework of society, medical culture, and relationship with family and friends. | Qualitative | The study identified themes, including mythical expectations, misconceptions about prenatal testing, and contradictory societal norms, creating dilemmas for women coping with loss. The paper includes suggestions for providers to assist with grief. | Provides insights into emotions of women who terminated pregnancy for fetal anomaly and the way social workers can assist patients as they process their grief. |
| McCoyd JL.   (2010) | USA | Practice | The implicit contract: Implications for health social work | Identify common patient dynamics, especially among women ending pregnancies due to fetal anomalies in the high-tech medical system and explore their relevance to health social work practice. | Qualitative | The data suggests that women frequently anticipate positive outcomes, particularly in advanced medical care for ensuring a healthy baby. The study stressed the significance of counseling provided by social workers in helping clients establish realistic expectations regarding pregnancy outcomes. | Explains importance of client counseling by social workers in setting realistic expectations from pregnancy outcomes. |
| McCoyd JL.  (2010) | USA | Policy | Women in no man's land: The abortion debate in the USA and women terminating desired pregnancies due to foetal anomaly | Qualitative exploration of how women made decisions about a pregnancy affected by foetal  anomaly, what they experienced in terms of attachment to their foetus and how their emotions unfolded after TFA. | Qualitative | Women are often surprised by the medical term "abortion" for ending a desired pregnancy, which, due to the polarized debate, isolates them from support. Despite pro-choice beliefs, they struggle to find a reference group in the politicized discourse. | Underlines the importance of social workers in advocating for abortion rights and supporting women who choose to terminate pregnancy due to fetal anomaly. |
| Murshid et al.  (2018) | USA | Research | Does the use of unreliable contraceptive methods increase the number of abortions? Results from a national survey of women in the United States | To assess the association between the types of contraceptive use (unreliable or traditional methods vs. modern methods) and  number of abortions among a nationally representative sample of abortion patients in the United States from the Abortion Patients Survey 2008. | Quantitative | 8.8% indicated that they never used contraceptives, 9% indicated using traditional methods, and 82.2% used modern methods.   Data revealed that women who used modern methods of contraception were more likely to have had previous abortions compared to women who did not use contraceptives and those who used traditional methods of contraception. | This article draws the significance of social workers in providing counseling and guidance to individuals on contraceptive methods. |
| Poehling et al.  (2023) | USA | Policy | From gaslighting to enlightening: Reproductive justice as an interdisciplinary solution to close the health gap | Explore how the Reproductive Justice framework guides interdisciplinary solutions to Social Work's Grand Challenges. Provide practical strategies for navigating emotional conversations in learning environments, fostering critical discussions on Reproductive Justice in social work practice. | Conceptual/Thought Piece | The Reproductive Justice (RJ) lens guides social workers in engaging with faith communities supporting abortion rights and applying intersectionality. It aligns with core social work values and is crucial for achieving environmental justice. Organizations like CSWE, NASW, ASWB, and social work educators should advocate for and teach the RJ framework. | Emphasize on teaching reproductive justice framework to social work students to develop skills of critical thinking and ethical decision making. |
| Pollack D  (2005) | USA | Practice | The capacity of a mentally challenged person to consent to abortion and sterilization | 1. Investigate historical discrimination against individuals with disabilities and the legal complexities of their informed consent in cases of abortion or sterilization.  2. Explore the role of social workers in bioethical decision-making. | Conceptual/Thought Piece | In different jurisdictions, abortion consent varies depending on the level of intellectual or developmental disability. Courts, not parents, usually have authority over consent in sterilization cases due to the long-term impact. In different jurisdictions, abortion consent varies depending on the level of intellectual or developmental disability. Courts, not parents, usually have authority over consent in sterilization cases due to the long-term impact. | Presents guidelines for social workers when dealing with clients with mental challenges when seeking abortion or sterilization. |
| Price SK.  (2008) | USA | Practice | Women and reproductive loss: Client-worker dialogues designed to break the silence | To synthesize theory and empirical research related to reproductive loss and subsequent parenting. | Conceptual/Thought Piece | Key dialogues discussed that can be used within social work practice to break silence for clients- Integrating Reproductive Loss into Assessment, Empowering Clients to Define Terms, Adaptive vs. Complicated Responses, Facing the Future. | Describes role of dialogues of reproductive loss within social work practice setting in breaking the silence for clients and for the social work profession. |
| Reamer F.  (2023) | USA | Practice | Ethical practice in a post-Roe world: A guide for social workers | To examine the Dobbs decision (which overturned Roe v. Wade), explores ethical challenges for social workers in reproductive health services, and provides guidelines for navigating ethical dilemmas. Emphasizes the significance of ethics-informed advocacy in reproductive health. | Conceptual/Thought Piece | Provides guidelines for social workers post Dobbs decision. Ways for protection of clients’ privacy and confidentiality, documentation and data sharing and ethics-based risk management protocols are discussed. | Examines social workers' challenges in safeguarding client confidentiality, documentation, and addressing client abandonment amidst stringent abortion laws. |
| Rosen et al.  (1974) | USA | Research | Health professionals' attitudes toward abortion | Analyze abortion attitudes from a 1971 nationwide survey among nursing, medicine, and social work students and faculty, comparing them with the general population's perspectives. | Quantitative | Social workers students (76%) expressed the highest positivity towards abortion and nurse faculty (49%) the least. | Describes attitudes of various health professionals towards abortion and various conditions under which health professionals would support abortion. |
| Sammons CC.  (1978) | USA | Practice | Ethical issues in genetic intervention | Discusses the psychosocial implications of genetic interventions, such as amniocentesis and selective abortion, including concepts like genetic health and privacy. It also touches on the role and training of social workers in assisting patients with these interventions. | Conceptual/Thought Piece | Social workers handling cases involving amniocentesis and selective abortion should be well-versed in legal issues such as outcomes of the interventions, informed consent, maintaining confidentiality related to genetic profile etc. | Focuses on ethical issues faced by social workers while working with patients choosing to get selective abortion. |
| Smith BD.  (2017) | USA | Policy | Reproductive Justice: A policy window for social work advocacy | To provide social workers with a concise understanding of reproductive rights and justice while summarizing threats across areas such as prevention, termination, pregnancy loss, personhood measures, and drug use during pregnancy. | Conceptual/Thought Piece | Social workers can contribute to the realm of reproductive rights and justice by identifying opportunities to influence policy, voicing their experiences gained as counselors, teaching social work ethics to students, and publishing research on topics related to reproductive justice | Reflects how social workers can stand for the rights of women and contribute to the area of reproductive rights and justice. |
| Smith-Osborne et al.  (2009) | USA | Research | Exploring the relationship between religiosity and political ideology among social workers | To examine social workers’ strength of religious/spiritual affiliation as it relates to political ideology. | Quantitative | Social workers with stronger religious ties opposed lesbian and gay rights and abortion rights. Political conservatives were less supportive of gay rights but favored the death penalty, while liberals were more supportive of abortion rights. | Explores the connection between social workers' stances on religiously influenced social issues, like abortion. |
| Sperlich et al.  (2020) | USA | Research | Reflections of stress in US abortion narratives | To generate new knowledge about the themes represented in abortion stories and about the experiences specific to   Southern, rural, Delta, and Appalachian U.S. populations. | Qualitative | The potential sources of stress were identified as legal restrictions, abortion stigma, encounters with antiabortion protesters and personal hardships. The study underscores the necessity for social work interventions tailored to alleviate stress throughout the process of seeking and obtaining an abortion. | Describes the need to apply the trauma-informed care framework across social work practice and with regard to abortion. |
| Streets F.  (2009) | USA | Education | Overcoming a fear of religion in social work education and practice | Examine social work educators' concerns about integrating religion into social work, emphasizing practitioners' need to consider clients' beliefs in addressing sensitive issues like homosexuality, divorce, end-of-life care, HIV/AIDS, and abortion while upholding social work ethics and values. | Conceptual/Thought Piece | Social workers ought to prioritize clients' beliefs when navigating sensitive topics such as abortion, placing them above their own personal convictions and biases. This method entails recognizing the significance of clients' core beliefs, understanding the societal influence of religion, and, if required, abstaining from or directing cases where a social worker's religious perspectives hinder their professional obligations. | Discusses the importance of understanding client’s religion in providing holistic social service on sensitive topics like abortion. |
| Strng KT.  (1978) | USA | Practice | Social work as an integral part of family planning service for low-income families: An example of U.S. experience | Explore social workers' role in addressing human problems within maternity and infant care for low-income families, focusing on service delivery methods and the significance for family planning among indigent mothers and pregnant teens. | Conceptual/Thought Piece | Family planning problems include abortion, adoption, aggressive-destructive behaviour, anxiety, child abuse or neglect, child-care need, discipline problem, emotional dysfunction, family planning need etc.   The study underscored the effectiveness of integrating family planning with social work support. | Role of social worker in providing family planning services including abortion. |
| Such-Baer M.  (1974) | USA | Practice | Professional staff reaction to abortion work | Explore staff members' emotional reactions to abortion work and identify influencing factors, examining the potential relationship between intellectual attitudes towards abortion and emotional responses to abortion work. | Quantitative | 58.5% of the respondents held pro-abortion intellectual attitudes. Social workers were least discomforted by abortion, physicians showed slightly more discomfort, and nurses were most discomforted by abortion work. The difference in emotional reaction by profession was significant at the .05 level. | Provides an insight into factors that cause emotional reaction of medical staff to abortion work. |
| Sung KT.  (1978) | USA | Education | Family planning services for indigent women and girls | To describe family planning services provided by social workers in a health  program for indigent mothers and their children and make recommendations for the future development of such programs. | Mixed Methods | Results suggested there are needs to collaborate with a social work school to address educational gaps, for regular evaluations and follow-ups ensure effective patient support, and understanding roles is crucial for clear referrals within clinics. Target women early, extending beyond pregnancy. Ongoing research is essential due to limited knowledge in social work practice for family planning. | Explains crucial role of social worker in counseling teenage mothers facing severe health or social problems like abortion. |
| Suslovic B.  (2018) | USA | Practice | Feminist social work and abortion support work: A young practitioner's reflections | To present a contrast between different approaches to care practiced in hospital systems in Boston, Massachusetts, and emphasis importance of feminist ethics of care for social work practice. | Conceptual/Thought Piece | Hospital care standards discourage integrating feminist ethics of care. The Boston Abortion Support collective, funded by donors, successfully integrates these ethics due to its collective management, lack of insurance billing, and client-centered policies. | Describes implications of feminist ethics of care approaches for feminist social work practice. |
| Tucker J.  (2011) | USA | Research | An exploratory study of sex selective abortion among Indian immigrants in the United States | To explore the transmission of and motivation for sex selective abortion among Indian immigrants in the United States. | Qualitative | Participants have direct and indirect exposure to sex-selective abortion and son preference, with television and movies being common sources. Themes from the analysis include issues related to girls, dowry, lack of education, familial pressure, preference, ties, inheritance, son's duty, money, name continuity, and the desire for a male child. | Underscores the need for social workers to be educated on cultural practices to comprehend their connection to individual behavior in the context of abortion. |
| Ullmann A.  (1995) | USA | Research | Social work service to abortion patients | To understand psychological implications of abortion on patients seeking abortion through group discussions. | Qualitative | Several concerns came up in the discussions including- feeling of guilt, ambivalent feelings of motherhood, attitude of the clinic personnel, feeling of isolation during the abortion process. | Describes ambivalent feelings of women around abortion and role of social worker in coping with any stress related to the abortion experience. |
| van Berkel C.  (2004) | Canada | Practice | Abortion work: Health care's best kept secret | An exploration of the experiences of individuals involved in abortion service to break the secrecy and silence that shroud abortion work and to increase recognition and support for both the work and the workers. | Qualitative | Abortion work involves cultural and institutional ambivalence, leading workers to adopt concealment strategies. Despite tensions and risks, workers express personal gratification and pride in their roles. | Describes inherent tensions in the abortion work faced by abortion providers, including social workers. |
| Winter et al.  (2016) | USA | Practice | Personal and professional values: Relationships between social workers' reproductive health knowledge, attitudes, and ethical decision-making | To explore how perceived abortion knowledge and anti-abortion attitudes are related to social workers' perceptions of ethical decision-making around reproductive health. | Quantitative | 82.3% disagreed with refusing service based on personal disagreement with a client's reproductive choice, but only 10.1% felt confident aiding a client seeking abortion information. Anti-abortion attitudes among students relate to reluctance in assisting disagreeable reproductive choices, while abortion-related knowledge ties to ethical decision-making in reproductive health. | Indicates that social work students' anti-abortion attitudes are significantly linked to their reported inability to assist clients considering reproductive health decisions they personally disagree with. |
| Witt et al.  (2022) | USA | Education | Examining social work students knowledge of and attitudes about abortion and curriculum coverage in social work education | Study social work students' awareness, knowledge, and attitudes on abortion, examining their feelings, awareness of the profession's stance, curriculum coverage, and exploring correlations between abortion knowledge and supportive attitudes towards abortion access. | Quantitative | Most social work students believe abortion should be legal in at least some circumstances (68.1%), and also that abortion laws should be less restrictive in the United States (52.7%). | Contributes to understanding abortion and reproductive rights within social work education. |
| Younes et al.  (2021) | USA | Education | A call to action: Addressing ambivalence and promoting advocacy for reproductive rights in social work education | To highlight the sociopolitical reality surrounding reproductive rights in the United States, and the ambivalence of social work education in integrating reproductive justice into the curriculum and professional preparation of students for practice. | Conceptual/Thought Piece | A call to action is made with a specific proposal for Council on Social Work Education and social work programs to consider for effective professional preparation of students on the topic of reproductive justice, including abortion. | Writes about importance of breaking silence on reproductive rights in social work through introduction of the related topic in curriculum, social action, and advocacy. |
| Zakus et al.  (1987) | USA | Practice | Adolescent abortion option | To highlight the vulnerability of adolescents to unwanted pregnancy and the need for specialized counseling services to support them in making decisions about abortion. | Conceptual/Thought Piece | Teens facing psychosocial challenges are at higher risk of unwanted pregnancies. Post-abortion, they grapple with challenges like body image, sexuality, and delayed help-seeking, intensifying their experiences. Social workers can play significant role through specialized counseling. This includes aiding in decision-making about abortion, assisting in coping with the procedure, and managing emotional challenges post-abortion. | Explains role of social work in adolescent counseling in the area of abortion. |
