# Supplementary material for: Understanding social work’s role in abortion care: A comprehensive scoping review
Source: PLoS One. 2025 Apr 24;20(4):e0320260. doi: 10.1371/journal.pone.0320260 (PMC12021202; doi:10.1371/journal.pone.0320260)
Supplement: S1 Appendix — Keyword search strategy used, illustrating APA PsychInfo (Ovid) search terms. (DOCX) [file pone.0320260.s002.docx]

**S1 Appendix A. Search Term and Strategy Examplar.** Keyword search strategy used, illustrating APA PsychInfo (Ovid) search terms.

1 exp social workers/

2 social work education/

3 exp social casework/

4 social services/

5 social work*.tw.

6 (case work* or casework*).tw.

7 social service*.tw.

8 social care.tw.

9 social welfare.tw.

10 (bsw or msw or rsw or lcsw).tw.

11 or/1-10

12 induced abortion/

13 reproductive rights/ or abortion laws/

14 "abortion (attitudes toward)"/

15 reproductive health/

16 reproductive health care/

17 abort*.tw.

18 (reproductive adj1 (justice or right* or health* or options or counseling or counselling or decision making)).tw.

19 (pregnan* adj2 (unwanted or terminat* or options or counseling or counselling or end)).tw.

20 (terminat* adj2 (medical or surgical)).tw.

21 miscarri*.tw.

22 pro-choice.tw.

23 pro-life.tw.

24 anti-choice.tw.

25 family planning.tw.

26 family planning/

27 or/12-26

28 11 and 27

29 limit 28 to yr="1973 -Current"
